# A baculovirus-mediated strategy for full-length plant virus coat protein expression and purification

**DOI:** 10.1186/1743-422X-10-262

**Published:** 2013-08-15

**Authors:** Daniel Mendes Pereira Ardisson-Araújo, Juliana Ribeiro Rocha, Márcio Hedil Oliveira da Costa, Anamélia Lorenzetti Bocca, André Nepomuceno Dusi, Renato de Oliveira Resende, Bergmann Morais Ribeiro

**Affiliations:** 1 Department of Cell Biology, Laboratory of Electron Microscopy, Institute of Biological Sciences, University of Brasília, Brasília, DF, Brazil; 2 Embrapa Vegetables, Embrapa, Brasília, Brazil

**Keywords:** Baculovirus expression system, Polyhedrin, Garlic virus coat protein, Virus-indexing diagnostic kit

## Abstract

**Background:**

Garlic production is severely affected by virus infection, causing a decrease in productivity and quality. There are no virus-free cultivars and garlic-infecting viruses are difficult to purify, which make specific antibody production very laborious. Since high quality antisera against plant viruses are important tools for serological detection, we have developed a method to express and purify full-length plant virus coat proteins using baculovirus expression system and insects as bioreactors.

**Results:**

In this work, we have fused the full-length coat protein (cp) gene from the Garlic Mite-borne Filamentous Virus (GarMbFV) to the 3′-end of the Polyhedrin (*polh*) gene of the baculovirus *Autographa californica multiple nucleopolyhedrovirus* (AcMNPV). The recombinant baculovirus was amplified in insect cell culture and the virus was used to infect *Spodoptera frugiperda* larvae. Thus, the recombinant fused protein was easily purified from insect cadavers using sucrose gradient centrifugation and analyzed by Western Blotting. Interestingly, amorphous crystals were produced in the cytoplasm of cells infected with the recombinant virus containing the chimeric-protein gene but not in cells infected with the wild type and recombinant virus containing the hexa histidine tagged *Polh*. Moreover, the chimeric protein was used to immunize rats and generate antibodies against the target protein. The antiserum produced was able to detect plants infected with GarMbFV, which had been initially confirmed by RT-PCR.

**Conclusions:**

The expression of a plant virus full-length coat protein fused to the baculovirus Polyhedrin in recombinant baculovirus-infected insects was shown to produce high amounts of the recombinant protein which was easily purified and efficiently used to generate specific antibodies. Therefore, this strategy can potentially be used for the development of plant virus diagnostic kits for those viruses that are difficult to purify, are present in low titers or are present in mix infection in their plant hosts.

## Background

For the establishment of a successful large-scale agricultural production, the use of healthy and pathogen-free plants is an essential measure. Garlic, for instance, has no virus-free cultivars, which represents a serious problem due to economic losses and the difficulties of controlling disease. Considering all agronomic parameters important for garlic production, bulb growth is the most severely affected by virus infections, causing a decrease in productivity and quality, with a reduction of up to 88% of the weight [1-3]. In fact, the introduction of the first virus-free cultivars produced in Brazil by thermotherapy and stem-tip culture has resulted in improved yields [4].

However, for the efficient production of tissue culture virus-free plants, some bottlenecks must be overcome. One of them is the detection of virus infections in garlic plants. Current detection is based on serological methods, using specific antiserum, symptomatology, transmission tests in different host plants, and sequence data of the coat protein gene [5,6]. In situations when a high number of samples need to be tested, the use of molecular diagnosis techniques, such as RT-PCR, is not an easy task, due to the requirement of expensive and fragile materials (e.g. enzymes, dNTPs, termocycler). Therefore, serological methods are recommended for large scale evaluations, however, high quality antisera are not available for all relevant viruses [7,8]. The dot-Enzyme-Linked Immunosorbent Assay (dot-ELISA) is a serological, less expensive and more practical alternative method that could be used to detect plant virus infections, despite of requiring the production of specific antibodies.

Many plant viruses of agricultural interest, including the garlic-infecting viruses, are difficult to purify from the host because they accumulate in low titers and are often present in mixed infections, which make specific antibody production very laborious [9-11].

One way of circumventing these difficulties is to express the virus coat protein in prokaryotic or eukaryotic cell systems for further antisera production. The expression of a soluble coat protein from a garlic virus in bacteria (Resende RO, personal communication) and insect cells [12] for production of antiserum for the recombinant protein was previously carried out by our research group but neither system worked. We were unable to produce high titer antibody due to problems in protein purification, which makes it unsuitable for the large-scale species-specific diagnosis of the tested garlic viruses. Despite baculovirus being a potent tool to express different proteins, the purification step is usually a challenge for further applications. To solve this problem, one could attempt to tag the recombinant protein with a carrier peptide or protein to facilitate the antigen purification.

Interestingly, the orally infective baculovirus virions are surrounded by protein crystal matrix composed mainly of a single protein, the Polyhedrin, which is highly expressed in the late stages of infection [13]. Polyhedrin has been used as a carrier protein to facilitate antigen purification [14-18]. This strategy for purification of antigen has been patented (see http://otl.sinica.edu.tw/en/index.php?t=9&group_id=19&article_id=477). The system has advantages when compared to existing technologies, it is both easy and cheap and achieves a purity of over 95% without the need for tags or expensive column purification steps. In this work we have expressed the coat protein of the *Allexivirus* GarMbFV (Garlic mite-borne filamentous virus) in caterpillars as bioreactors using a Polyhedrin-based expression vector in order to generate polyclonal antibodies for a potential development of a large-scale dot-ELISA-mediated virus-indexing diagnostic kit.

## Results

### Fusion vectors and recombinant virus construction

**Figure 1:**
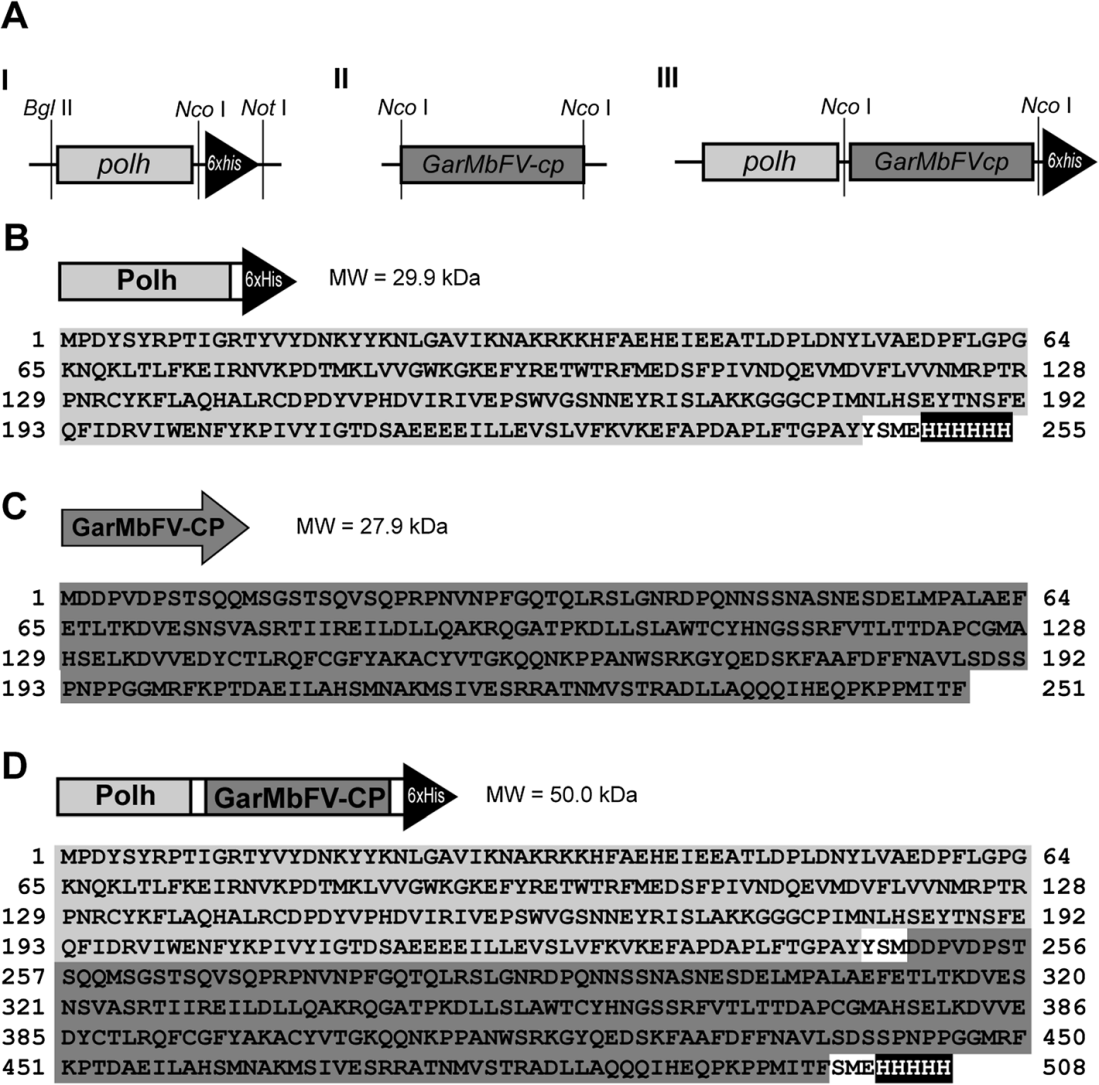
**Gene and protein schemes with deduced amino acid sequence. (A)** The *polh-6xhis* fragment was amplified and cloned into the commercial vector (I), pFB1 to generate pFB1-*polh-6xhis* (not shown). We used *Bgl*II (primer added) and *Not*I (from the pGem-T® easy vector) restriction sites to clone the modified gene and *Nco*I (primer added) restriction site to use for virus coat protein fusion (II and III). These vectors were used to construct recombinant viruses, vAc-*polh*-*6xhis* and vAc-*GarMbFV-cp*-*polh*-*6xhis* by site-specific transposition in *E. coli* (Bac-to-bac® system, Invitrogen). The virus expressing non-fused GarMbFV-CP was constructed by homologous recombination inside insect cells co-transfected with DNA from pSyn-*GarMbFV-cp* and vSynVI-gal (see Methods). Deduced amino acid sequence of the **(B)** non-fused coat protein, GarMbFV-CP (27.9 kDa), **(C)** Polh-6xHis (29.9 kDa), and **(D)** Polh-GarMbFV-CP-6xHis recombinant protein (50.0 kDa) are shown.

In order to express a chimeric protein containing the GarMbFV coat protein fused to the AcMNPV *polh*gene, a shuttle vector, pFB1-*polh-6xHis* with a modified *polh* gene was constructed (Figure 1). This modified ORF shows a unique *Nco*I restriction site for fusion in frame of any gene at the 3′-end and also six histidine codons for recombinant protein immunoblotting identification (Figure 1A-I and B). The *GarMbFV* coat protein gene was amplified (Figure 1A-II and C) and inserted into the modified *polh* gene to generate the plasmid pFB1-*polh-GarMbFV-cp-6xHis* (Figure 1A-III). The modified vector presented a new ORF containing the fusion protein Polh-GarMbFV-CP-6xHis (Figure 1D). Both derived vectors were used to construct the recombinant viruses, vAc-*polh-6xhis* and vAc-*polh-GarMbFV-cp-6xhis* by the Bac-to-bac system (Invitrogen). The recombinant viruses were amplified in insect cells and confirmed by PCR analysis (not shown). Furthermore, a donor vector was constructed for homologous recombination to generate an engineered virus expressing the non-fused *GarMbFV-cp*. This soluble protein was expressed under the control of a late and a very late promoter *in tandem* (Wang et al., 1991) present in the recombinant vector pSyn-*GarMbFV-cp*.

### Recombinant protein analysis

**Figure 2:**
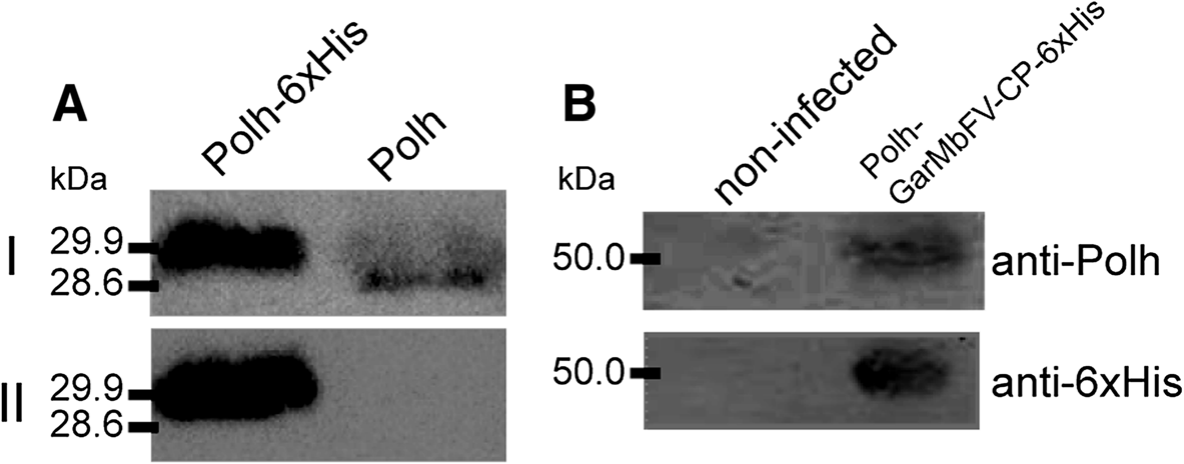
**Expression analysis of wild type Polyhedrin and recombinant proteins.** AcMNPV-, vAc-*polh-6xhis*- and vAc-*polh-GarMbFV-cp-6xhis*-infected Tn5B extracts were separated by 12% SDS-PAGE (not shown) and the proteins transferred to nitrocellulose membranes. The membranes were then treated with specific antibodies anti-Polh (I – upper panels in **A** and **B**) and anti-6xHis (II – lower panels in **A** and **B**). The anti-Polh detected the wild type Polyhedrin as well as the recombinant protein fused to Polyhedrin. On the other hand, the anti-6xHis detected only the recombinant proteins.

Synthesis of recombinant proteins was analyzed by immunoblotting. Virus-infected Tn5B extracts were separated by 12% SDS-PAGE (not shown) and the proteins transferred to a nitrocellulose membrane. The proteins were detected using anti-hexa-histidine antibody (anti-6xHis) and anti-Polh antiserum (anti-Polh) (Figure 2). An immunoreactive band of 29.9 kDa was detected in extracts of vAc-*polh-6xHis*-infected cells (72 h p.i.) when using anti-Polh antiserum (Figure 2A, I) and anti-6xHis (Figure 2A, II). No bands were detected in non-infected cell extracts (not shown). Further, when extracts from AcMNPV-infected cells were tested with the same antibodies, just one band reacted with the anti-Polh, showing a smaller mass of 28.6 kDa (Figure 2A) as expected. Additionally, extracts containing the chimeric protein Polh-GarMbFVcp-6xHis were probed with anti-Polh and anti-6xHis showing a 50 kDa immunoreactive band, thus revealing correct recombinant protein fusion and expression. No band was detected in mock-infected cell extracts (Figure 2B).

### Light microscopy analysis of cells infected with recombinant viruses

**Figure 3:**
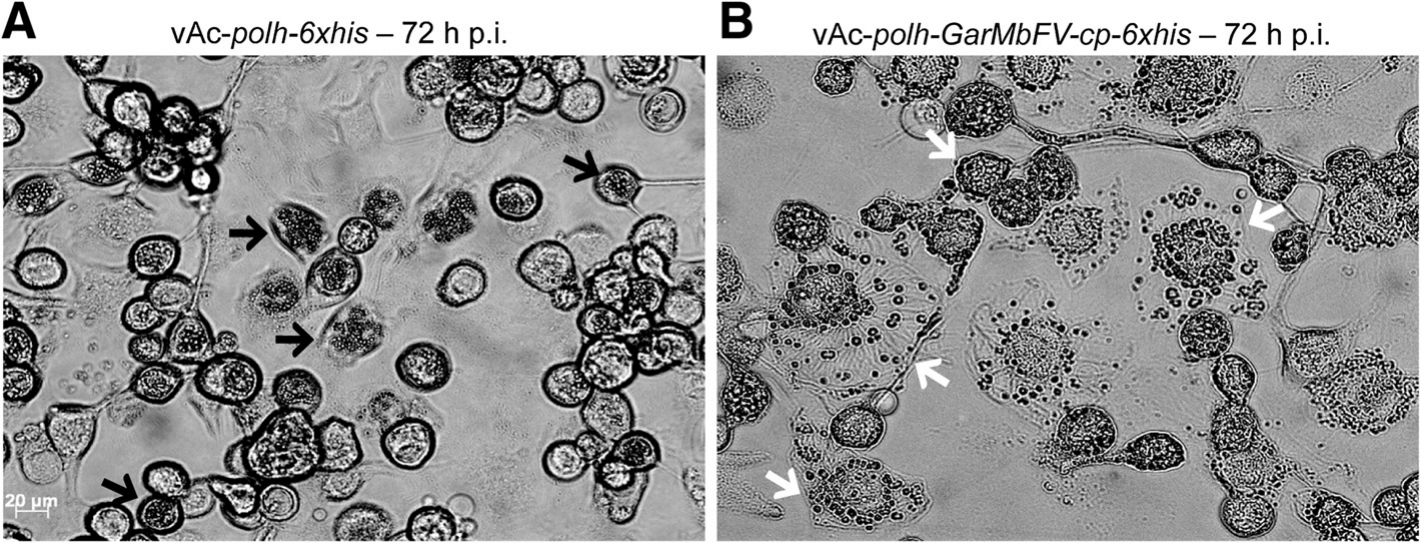
**Structural analysis of both vAc-*polh-6xhis*- and vAc-*polh-GarMbFV-cp-6xhis*-infected Tn5B cells at 72 h p.i. (A)** vAc-*polh-6xhis-*infected cells showing the presence of numerous occlusion bodies inside the cell nucleus (black arrows). **(B)** vAc-*polh-GarMbFV-cp-6xhis*-infected Tn5B cells showing the occlusion bodies derived from the recombinant fused protein mainly in the cytoplasm of the cells (white arrows). Scale bar = 20 μm.

vAc-*polh-6xhis* and vAc-*polh-GarMbFV-cp-6xhis*-infected Tn5B cells (72 h p.i.) were analyzed by light microscopy. Occlusion bodies resembling wild type polyhedra were detected in the nucleus of vAc-*polh-6xhis*-infected cells (black arrows, Figure 3A). When cells were infected with vAc-*polh-GarMbFV-cp-6xhis*, it was possible to see irregular shaped crystals mainly in the cells’ cytoplasm (white arrows, Figure 3B).

### Analysis of purified recombinant crystals

**Figure 4:**
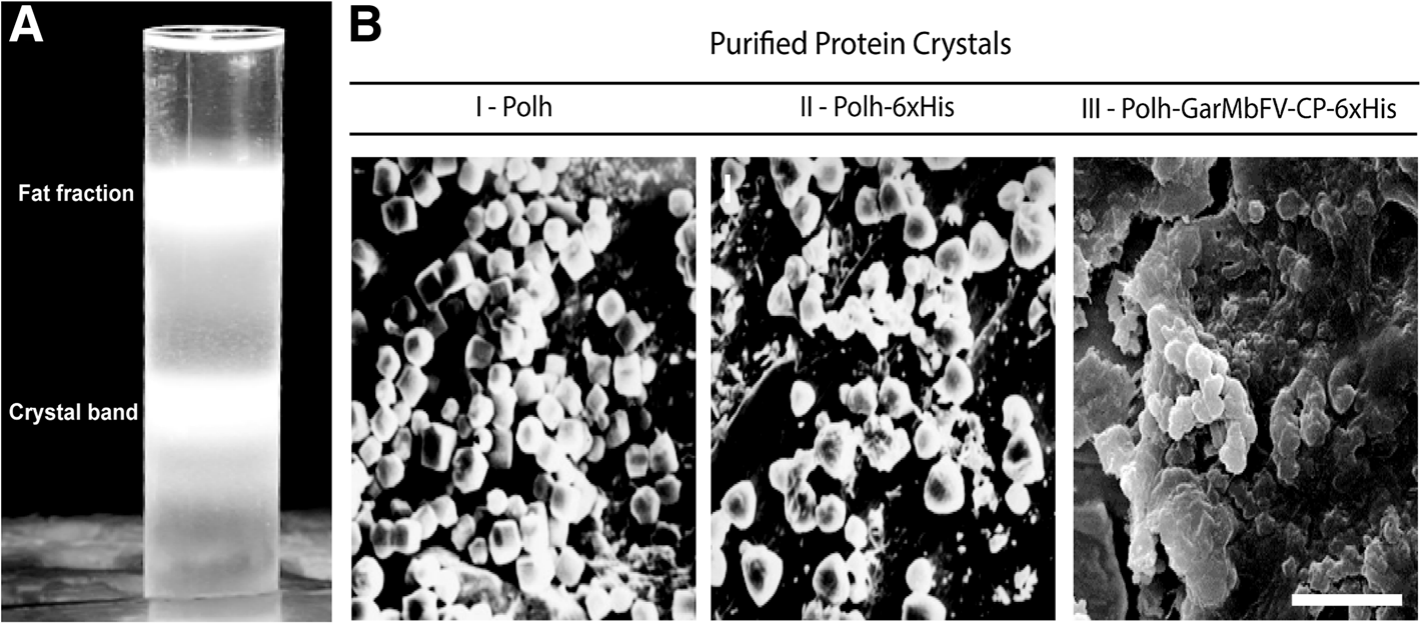
**Purification and ultrastructural analysis of occlusion bodies derived from wild type and recombinant viruses infected insects.** AcMNPV polyhedra (Polh) and Polh-6xHis and Polh-GarMbFV-CP-6xHis crystals from infected *S. frugiperda* cadavers were purified by centrifugation through a sucrose gradient. **(A)** A centrifuge tube after centrifugation of vAc-*polh-GarMbFV-cp-6xHis*-infected insect extracts showing a lower band containing the putative crystals. The upper band shows the fat fraction from the insect cadavers. **(B)** The crystals were collected from the gradient and prepared for scanning electron microscopy: (I) Occlusion bodies from wild type AcMNPV infected insect cadavers; (II) Occlusion bodies from vAc-*polh-6xhis*-infected insect cadavers showing a triangular shaped crystal; and (III) Occlusion bodies-like from vAc-*polh-GarMbFV-cp-6xhis*-infected insect cadavers showing some crystals and undefined mass protein of Polh-GarMbFV-CP-6xHis (Scale bar = 5 μm).

Purified occlusion bodies from wild type and recombinant virus-infected *S. frugiperda* larva were analyzed by scanning electron microscopy. All occlusion bodies formed a distinct band on the sucrose gradient (Figure 4A). AcMNPV occlusion bodies showed a regular cubic shape as expected (Figure 4B-I), on the other hand, the vAc-*polh-6xhis* showed mainly triangular shaped occlusion bodies (Figure 4B-II) and the vAc-*polh-GarMbFV-cp-6xhis* showed putative occlusion bodies of amorphous shape (Figure 4B-III).

### Antiserum production and identification of GarMbFV-infected garlic plants

**Figure 5:**
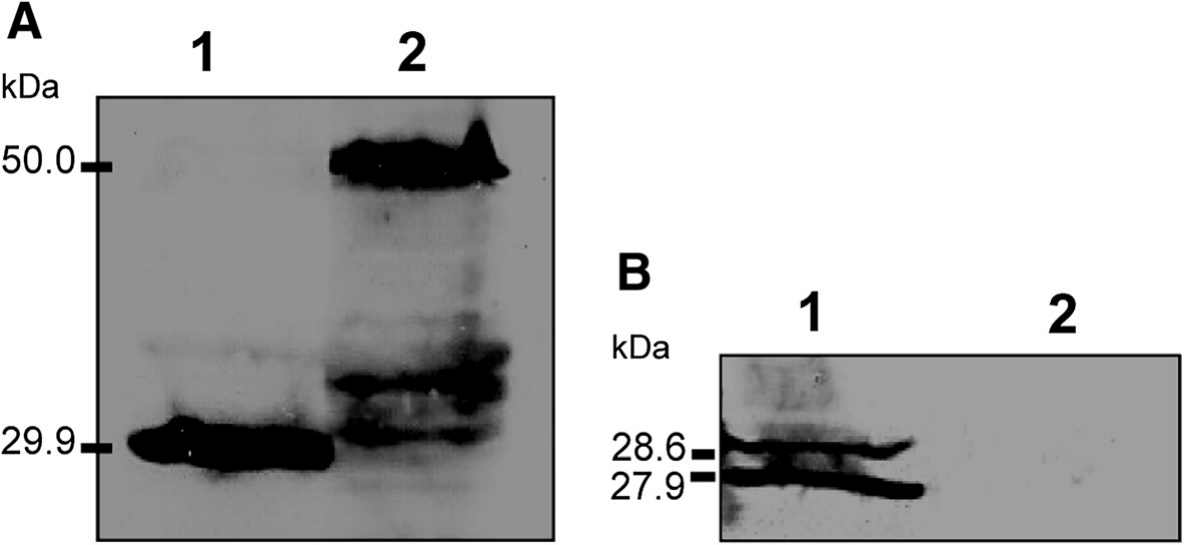
**Immunoblot against infected- and non-infected-insect cell extracts using anti-Polh-GarMbFV-CP-6xHis.** vAc-*polh-6xhis*-, vAc-*polh-GarMbFV-cp-6xhis*-, and vSyn-*GarMbFV-cp*-infected Tn5B extracts were separated by 12% SDS-PAGE (not shown) and transferred to nitrocellulose membrane. The membranes were treated with the antiserum anti-Polh-GarMbFV-CP-6xHis crystals. **(A)** Polh-6xHis (lane 1) and chimeric protein Polh-GarMbFV-CP-6xHis (lane 2). **(B)** Soluble GarMbFV-CP and Polh from vSyn-*GarMbFV-cp*- (lane 1) and non-infected cells extract (lane 2). Lower bands in lane 2 of figure A are most probably degradation products of the Polh-GarMbFV-CP-6xHis protein.

The purified fusion protein Polh-GarMbFV-CP was solubilized and used to immunize rats. The antiserum was tested in extracts derived from infected insect cells and garlic plants with visible virus infection symptoms (mosaic) by SDS-PAGE/immunoblotting and Dot-ELISA technique, respectively. Virus-infected Tn5B extracts were separated by 12% SDS-PAGE (not shown) and transferred to a nitrocellulose membrane. Immunoreactive bands of 29.9 and 50.0 kDa were detected in extracts of vAc-*polh-6xhis*- and vAc-*polh-GarMbFV-cp-6xhis*-infected cells (72 h p.i.) using anti-Polh-GarMbFV-CP-6xHis antiserum (Figure 5A, lane 1). Furthermore, the antiserum produced was tested against vSyn-GarMbFV-cp- and mock-infected extract cells. Two immunoreactive bands were found in the first extract, one of 29.9 kDa, the molecular weight of the Polyhedrin and another one of 27.9 kDa, the GarMbFV-CP weight, as expected. The specificity of the antiserum produced against insect cell was checked and there was no detection of any protein in mock-infected cell extracts (Figure 5A, lane 2).

**Figure 6:**
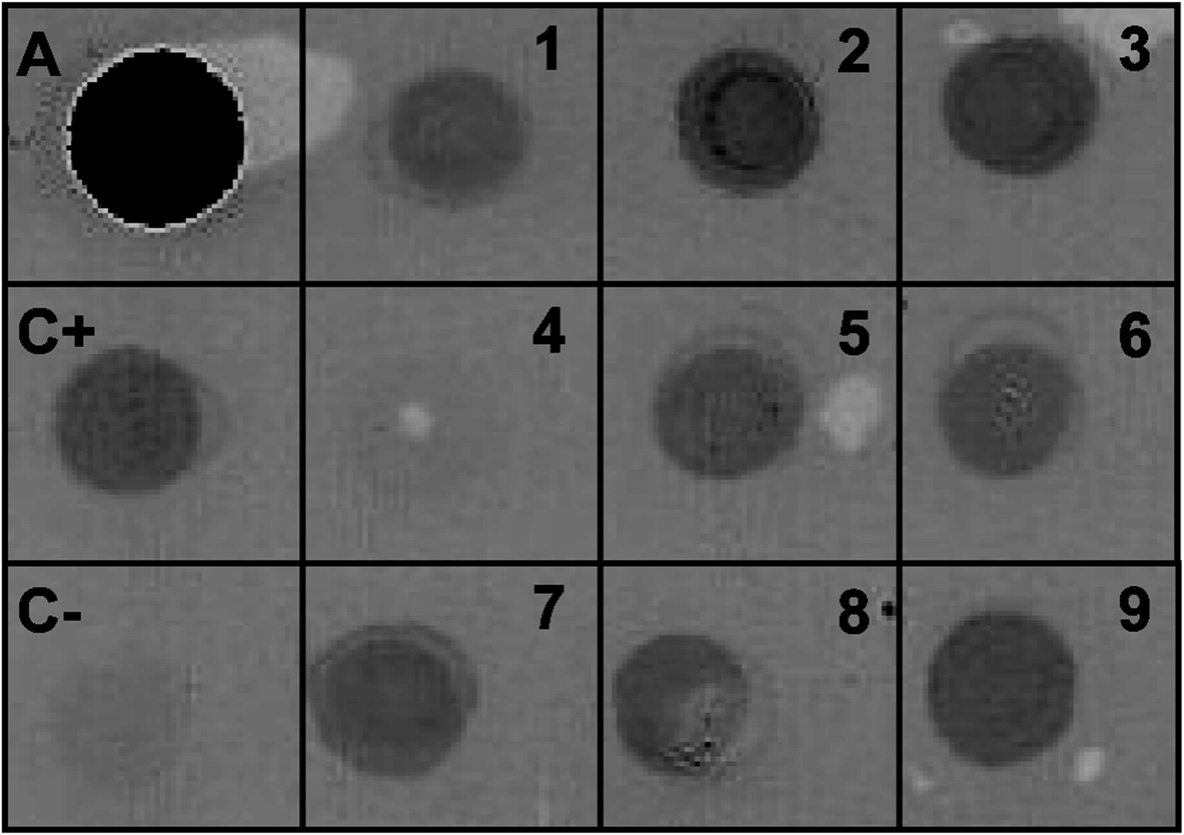
**Dot-ELISA of garlic leaf extracts.** A shows the antigen (baculovirus-expressed GarMbFV-CP) used to obtain the antiserum, (C+) shows a positive control derived from a GarMbFV-infected leaf extract confirmed by RT-PCR (not shown), in (C-) a virus-free garlic leaf extract as negative control. Nine extracts (1 to 9) from different plants showing virus infection symptoms were denaturated with modified Laemmili’s buffer, manually dotted, and tested with the antiserum obtained in this work. Only sample 4 did not react with the antiserum produced.

For dot-ELISA, the purified fusion protein (A), both GarMbFV-infected (C+) and uninfected (C-) garlic leaf extracts were used as controls (Figure 6 square A, C+, and C-). Garlic leaf extracts from nine different plants showing symptons of virus infection reacted with the produced antiserum (Figure 6, numbers 1 to 3 and 5 to 9). On the other hand, the extract number 4, even presenting symptoms, did not react with the antiserum (Figure 6). RT-PCR was carried out to corroborate the dot-ELISA results. The same positive and negative results were observed by the serological technique (not shown).

## Discussion

Brazil produces less garlic (*Allium sativum*) than it consumes, even with a local production reaching 140,000 t in 2011 (Brazilian Institute of Geography and Statistics - http://www.ibge.gov.br). Sixty percent of the total Brazilian fresh or chilled garlic imports are from China (Ministry of Development, Industry, and Foreign Trade - http://www.desenvolvimento.gov.br), and there is no phytosanitary barriers to garlic import and other plants, making the introduction of infected plants with potentially new pathogens a constant threat [19,20]. Utilizing a garlic virus coat protein as a model, we proposed the use of caterpillars as bioreactors and Polyhedrin as a carrier protein to facilitate the large-scale purification of full-length recombinant proteins of significant agricultural importance.

Polyhedrin-based amorphous crystals carrying the complete coat protein (cp) from GarMbFV in virus-infected insect cells and larvae were successfully produced. The incorporation of GarMbFV-CP into the polyhedra-like amorphous crystal depended on the structure of the new chimeric protein generated, which prevents the natural form, nuclear occlusion body formation and therefore, no virion incorporation into the occlusion body. Roh et al. [16] hypothesized that interactions between chimeric Polyhedrins can occur, but these protein masses are not as compact as the wild-type polyhedra. Ji et al. [21] resolved the structure of the AcMNPV polyhedra revealing a highly symmetrical covalently cross-braced robust lattice with flexible adaptors for virion occlusion. The Polyhedrin subunit may be broken down into three parts: N-terminal head, a β-barrel body, and C-terminal tail [21]. Here, we fused the complete GarMbFVcp gene in the Polyhedrin C-terminal tail without a proteolytic site. This tail is able to form a hook, jutting outwards which interacts with another Polyhedrin to form the crystal. Thus, the amorphous-shaped protein mass observed by scanning microscopy compared to the wild-type and the 6x-His-tagged Polyhedrin (Figure 4B), is probably due to both the size of the fused gene and the c-terminal fusion with the Polyhedrin protein. This feature avoided the polyhedral matrix formation and the nuclear localization by the chimeric protein. Previous research has used, besides the *polh* gene fused with a gene of interest, a second *polh* gene copy [14,17]. Although the presence of a second copy of *polh* could improve the chimeric crystal formation and nuclear localization, we observed that the presence of only one fused Polyhedrin-copy was sufficient to form a crystal structure. This allows recombinant protein purification from insect cadavers and cells as previously observed *in vitro*[16]. The purification of the recombinant protein is carried out by using sucrose gradient centrifugation which is both easier and cheaper than column chromatography which is normally used for recombinant protein purification.

The expression of the full-length fusion protein produced a polyhedra-like crystal that presented altered occlusion body morphology and localization, but this alteration did not affect protein purification in our study. Purified crystals carrying the GarMbFVcp showed that the coat protein antigens elicited the production of antibodies in rats. The results indicated that immunization with a chimeric protein induced a significant serum antibody reaction against Polyhedrin and the coat protein. This demonstrated that both were immunogenic and that the baculovirus protein perhaps increases the immune response as previously observed [17,22] for another baculovirus carrier protein and the Polyhedrin. Similar methods have already been developed and employed to purify target proteins [16-18]; however, the novelty presented here is the expression of a full-length protein in fusion with the Polyhedrin and the use of complete cadavers of caterpillars as bioreactors.

Unlike what was observed by Alves-Junior et al. [12], the antiserum produced by this new approach using the Polyhedrin as a full-length protein carrier was able to detect infected plants in dot-ELISA assays and to distinguish infected from non-infected plants at a high antiserum dilution (1:1,000 from the crude antiserum) and this detection was confirmed by RT-PCR using specific GarMbFV-cp primers (data not shown).

A challenge for a virus-indexing diagnosis in the field is the fact that this particular garlic disease is caused by a viral complex [20]. In Brazil alone, three virus genera related to garlic mosaic symptoms [3] were found: *Potyvirus* [*Onion yellow dwarf virus* (OYDV) and *Leek yellow stripe virus* (LYSV)] [23], *Carlavirus* [*Garlic common latent virus* (GCLV) and *Shallot latent virus* (SLV)] [23,24], and *Allexivirus*, [*Garlic miteborne filamentous virus* (GarMbFV), *Garlic virus C* (GarV-C) and *Garlic virus D* (GarV-D)] [8,25]. Notably, a symptomatic plant was found to be negative for GarMbFV, in both dot-ELISA and RT-PCR tests, suggesting that the produced antiserum did not cross-react to other viruses in the complex, although more experiments are necessary to confirm this result. Moreover, indirect ELISA or sandwich ELISA kits based on our strategy can be also developed.

## Conclusions

The expression of a plant virus full-length coat protein gene fused to the baculovirus Polyhedrin in recombinant baculovirus-infected insects was shown to produce high amounts of the recombinant protein which was easily purified and efficiently used to generate specific antibodies. Therefore, this strategy could be used for the development of plant virus diagnostic kits of those viruses that are difficult to purify, are in low titers, or are present in mix infections in their plant hosts.

## Methods

### Insect cells, viruses and insects

*Trichoplusia ni* (cabbage looper) BTI-Tn5B1-4 (Tn5B) cells [26] were maintained in TC-100 medium (HIMEDIA) supplemented with 10% fetal bovine serum (Invitrogen), and an antibiotic-antimycotic mixture (Gibco) at 28°C. Wild type *Autrographa californica multiple nucleopolyhedrovirus* (AcMNPV); vSynVI-gal [27] (an AcMNPV recombinant which contains the β-galactosidase (lac-Z) gene in place of the *polh* gene); recombinant viruses vSyn-*GarMbFV-cp*, vAc-*polh-GarMbFV-cp-6xhis* and, vAc-*polh-6xhis* constructed in this work have been propagated and their titers determined according to O’Reilly et al. [28]. *Spodoptera frugiperda* larvae, the fall armyworm, in early five-instar was provided by EMBRAPA/CENARGEN – Genetic Resources and Biotechnology (Brasília, Brazil), maintained at 25°C, and fed on an artificial diet [29]. The infection was carried out by injection of 10 μl of medium containing recombinant virus (10^6^ viruses in BV phenotype) into the hemocoel.

### Coat protein and *polh* amplification

The GarMbFV coat protein (GarMbFVcp) [25] (Genbank accession number X98991) was amplified using the F-GarMbFVcp^N^ (*CCA TGG* ACG ACC CTG TTG ACC CAA GC) and R-GarMbFVcp^N^ (*CCA TGG* AGA ACG TAA TCA TGG GAG G) oligonucleotides that modify the stop-codon and add *Nco*I restriction sites (in italics) flanking the gene for posterior fusion. The AcMNPV *polh* gene was modified by PCR in order to construct the fusion vector. Forward primer (Acpol-BglII F) adds a *Bgl*II restriction site (in italics) (CCG *AGA TCT* ATG CCA GAT TAT AGC TAT AGG CC) at the 5′-terminus and the reverse primer (Ac-pol/hisc NcoI R) removes the gene stop-codon and adds a *Nco*I restriction site (in italics), and six histidines codons (underlined sequence) at the 3′-terminus (TTA GTG ATG ATG ATG ATG ATG TT*C CAT GG*A ATA ATA CGC GGG GCC GGT AAA CAG AGG TGC). The PCR program used for both reactions was: 94°C/5 min, 35 cycles of 94°C/s, 50°C/20 s and 72°C/40 s and a final extension for 7 min at 72°C. Reactions contained 10 ng of DNA-sample, 300 μM of dNTP mix (Fermentas), 0.4 μM of each set of primers previously described, and the LongAmp enzyme (New England Biolabs). The modified fragments obtained (*GarMbFV-cp* and *polh-6xHis*) were analyzed by electrophoresis in 0.8% agarose gels [30], eluted using the GFX PCR DNA and Gel Band Purification kit (GE Healthcare), cloned into the pGem®-T easy vector (Promega), and sequenced (Macrogen, Korea) to certify the modifications.

### Fusion vector and recombinant virus construction

The *polh-6xhis* gene was removed from the cloning vector by *Bgl*II and *Not*I restriction digestion following the manufacturer’s instructions (Promega) and analyzed by electrophoresis in a 0.8% agarose gel [30]. The DNA fragment was then purified using the GFX™ PCR DNA and Gel Band Purification Kit (GE Healthcare) and cloned into the commercial donor vector pFastBac1® (pFB1 – Invitrogen) previously digested with *Bam*HI and *Not*I (Promega) restriction enzymes in order to generate the recombinant plasmid pFB1-*polh-6xhis*. The *Nco*I-flanked *GarMbFV-cp* gene, was removed from the pGem-T easy vector by *Nco*I restriction digestion, the fragment was analyzed by electrophoresis and purified from the gel as described above. The purified fragment was then introduced into the unique *Nco*I site present in the pFB1-*polh-6xhis* plasmid in order to construct the pFB1-*polh-GarMbFV-cp-6xhis* plasmid. Both vectors (pFB1-*polh-6xhis* and pFB1-*polh-GarMbFV-cp-6xhis*) were transformed into DH10-Bac cells (Invitrogen) by electroporation [30] and recombinant bacmids were selected following the manufacturer’s instructions (Bac-to-Bac®, Baculovirus expression systems, Invitrogen). DNA from the bacmids were purified and the presence of the recombinant gene was checked by PCR using specific oligonucleotides as described by the manufacturer’s protocol (Invitrogen). One microgram of each recombinant bacmid was transfected into Tn5B cells (10^6^) using liposomes (Cellfectin®) according to manufacturer’s instructions (Invitrogen). Since naked baculovirus DNA is infectious, the supernatant of 7 days post-transfection Tn5B cells containing the recombinant viruses were collected, tittered, and the virus amplified by infection of 1,5 × 10^7^ cells with a MOI of 1 in 75 cm^2^ flasks (TPP) as described in O’Reilly et al. [28].

For the construction of a recombinant virus containing the coat protein gene, the previously described plasmid, pGem-*GarMbFV-cp*[25], was digested with *Eco*RI (Promega) and analyzed by electrophoresis in a 0.8% agarose gel [30]. The DNA fragment was purified as previously described and cloned into the unique *Eco*RI restriction site of the transfer vector pSynXIVVI + X3 [27], which enables insertion of the heterologous gene under the control of two strong promoters *in tandem* (pSyn and pXIV). The vector pSynXIVVI + X3 had been previously digested with *Eco*RI (Promega) and dephosphorilated using SAP (Shrimp Alkaline Phosphatase) enzyme (Promega). One microgram of the resulting recombinant plasmid was co-transfected with 0.5 μg of the viral DNA from vSynVI-gal [27] in Tn5B cells (10^6^) using liposomes (as described above). Seven days after co-transfection, the cell supernatant was collected and used for recombinant virus isolation by serial dilution in 96-well plates [28]. The recombinant virus was amplified by infection of Tn5B (1.5 × 10^7^ cells) with an MOI of 1 in 75 Cm^2^ flasks (TPP).

### Putative recombinant protein crystals purification

Wild type and recombinant viruses were used to infect 200 *S. frugiperda* larvae each as described above. After the death by infection, the cadavers were homogenized with the same volume of ddH_2_O (w/v), filtered through gauzes and centrifuged at 7,000 *x g* for 10 min. The supernatant was discarded and the pellet was resuspended in the same volume of 5% Triton X-100 and centrifuged at 7,000 *x g* for 10 min. This procedure was repeated twice. The last pellet was resuspended in 0.5 M NaCl, centrifuged once more as above, and resuspended with ddH_2_O. All solutions contained a Protease Inhibitor Cocktail (Sigma – according to notes of use). The suspended solution was loaded on a discontinuous sucrose gradient (40-80% of sucrose in Phosphate Buffered Saline [PBS], 137.0 mM NaCl, 2.7 mM KCl, 10.0 mM Na_2_HPO_4_, 2.0 mM KH_2_PO_4_, pH 7.4) and centrifuged at 130,000 *x g* for 3 h. The band containing the putative crystals were removed from the gradient, five-fold diluted with ddH_2_O, and centrifuged at 7,000 *x g* for 10 min. Cadavers of vAc-*polh-6xhis*-infected larva were purified according to standard method for polyhedra purification [28]. The purified crystals were subjected to SDS-PAGE and ultrastructural analysis.

### Microscopy analysis

A monolayer of Tn5B cells (5.0 × 10^6^) was infected at an MOI of 5 with vAc-*polh-GarMbFV-cp-6xhis* and vAc-*polh-6xhis*. The infected cells were observed and photographed at 72 h p.i. in an Axiovert 100 inverted light microscope (Zeiss). For scanning electron microscopy (SEM), the purified putative crystals were dried in a critical point-dried (Balzers) and coated with gold in a Sputter Coater (Balzers) before being observed in a SEM Jeol JSM 840A at 10 kV.

### Analysis of recombinant protein synthesis

Tn5B cells (5.0 × 10^6^) were infected (10 pfu/cell) with vSyn-*GarMbFV-cp*, vAc-*polh-GarMbFV-cp-6xhis*, vAc-*polh-6xhis*, and the wild-type AcMNPV. At 72 h p.i., the infected cells were collected and centrifuged at 10,000 *x g* for 2 min. The resulting pellets were resuspended in PBS with an equal volume of 2x protein loading buffer (0.25 M Tris-Cl, pH 6.8, 4% SDS, 20% glycerol, 10% 2-mercaptoethanol, and 0.02% bromophenol blue). Proteins were heated (100°C) for 5 min and analyzed by 12% SDS-PAGE using the Mini Protean Tetra Cell aparatus (BioRad) following the manufacturer’s instructions. For immunoblotting, samples were resolved by 12% SDS-PAGE and transferred onto a nitrocellulose membrane (Sigma) using the Trans-Blot® SD – Semi Dry Transfer Cell (BioRad). The membrane was then blocked in 1× PBS Buffer (137 mM NaCl, 2.7 mM KCl, 10 mM Na_2_HPO_4_, 2 mM KH_2_PO_4_, pH 7.4) containing 3% skimmed milk powder for 16 h at 4°C, washed three times with PBS tween (0.05%) and probed with (i) mouse monoclonal anti-hexa-histidine (anti-6xHis) antibody (Sigma), (ii) rabbit anti-Polh antiserum [31], or (iii) anti-Polh-GarMbFV-CP-6xHis (produced in this work) followed by incubation with the alkaline phosphatase-conjugated anti-mouse/rat or anti-rabbit secondary antibodies (Sigma). Blots were developed using the NBT/BCIP (Sigma) substrate dissolved in alkaline phosphatase buffer (NaCl 100 mM, MgCl_2_ 5 mM e Tri-HCl 100 mM – pH 9.0).

### Immunization and antiserum production

The purified recombinant crystals of Polh-GarMbFV-CP-6xHis was dissolved for 1 h at 37°C in 0.1 M of Na_2_CO_3_, neutralized with 0.1 M of Tris–HCl (pH 7.6) and centrifuged at 16,000 *x g* for 30 min. The protein concentration was estimated by SDS-PAGE, comparing different BSA concentrations with the query protein (data not shown). The solution was filter sterilized and used for quadriceps intramuscular injection firstly with Freund’s complete adjuvant at 1:1 proportion, secondly with Freund’s incomplete adjuvant at 1:1 proportion, and finally alone. Five Gnotobiotic Sprague–Dawley male rats strain CD, 8 weeks old with food and water *ad libidum*, were used in this immunization experiment. The project was reviewed by the Ethics Committee on Animal Use of the University of Brasília. The animals were immunized with 15 days intervals for each injection containing 500 μg of the recombinant protein.

### Dot enzyme-linked immunosorbent assay (dot-ELISA)

Garlic plants infected with Garlic Mite-borne Filamentous Virus (GarMbFV) showing typical symptoms on the leaves such as yellow mosaic, stripes, and distortion and a general plant growth reduction were collected in the garlic germplasm bank from Embrapa Vegetables (Brasilia-DF, Brazil). The garlic leafs were triturated in PBS using a proportion of 100 μl of PBS per each 100 mg of leaves. The samples were mixed with equal volumes of 2X protein loading buffer without stain and heated (100°C) for 5 min. Purified antigen (Polh-GarMbFV-CP-6xHis), virus-infected, and non-infected leaf samples were manually dotted on a nitrocellulose membrane and probed against the crude rat anti-Polh-GarMbFV-CP-6xHis antiserum at 1:100, 1:500 and 1:1,000 (v/v) dilutions in 1X PBS plus 0.5% BSA (w/v) followed by incubation with the alkaline phosphatase-conjugated mouse secondary antibodies (Sigma) and NBT/BCIP substrate (Sigma) in alkaline phosphatase buffer. The positive and negative control plant extracts were confirmed by RT-PCR according to Fayad-André et al. (2011).

## Competing interests

The authors declare that they have no competing interests.

## Authors’ contributions

Conceived and designed the experiments: DMPAA, BMR, ROR. Performed the experiments: DMPAA, JRR, MHOC, Analyzed the data: DMPAA, BMR, ROR. Contributed reagents/materials/analysis tools: BMR, ROR, ALB, AND. Wrote the paper: DMPAA, BMR, ROR. All author s read and approved the final manuscript.
